# Children and Adolescent Mental Health in a Time of COVID-19: A Forgotten Priority

**DOI:** 10.5334/aogh.3330

**Published:** 2021-06-29

**Authors:** Agnes Binagwaho, Joyeuse Senga

**Affiliations:** 1 University of Global Health Equity, Kigali, Rwanda

## Abstract

Globally, 10–20% of children and adolescents experience mental health conditions, but most of them do not receive the appropriate care when it is needed. The COVID-19 deaths and prevention measures, such as the lockdowns, economic downturns, and school closures, have affected many communities physically, mentally, and economically and significantly impacted the already-neglected children and adolescents’ mental health. As a result, evidence has shown that many children and adolescents are experiencing psychological effects such as depression and anxiety without adequate support. The consequences of not addressing the mental health conditions in children and adolescents extend through adulthood and restrict them from reaching their full potential. The effects of COVID-19 on children and adolescents’ mental health highlight the urgent need for multisectoral home-grown solutions to provide early diagnosis and treatment and educate caregivers on home-based interventions and community outreach initiatives to address children and adolescents’ mental health challenges during this pandemic and beyond.

In 2015, the Sustainable Development Goals recognized mental health as a critical component of the global health agenda [1]. The World Health Organization (WHO) estimates that 10–20% of children and adolescents experience mental health conditions, such as depression, anxiety, and behavioral disorders, that are not diagnosed and treated in a timely manner [2]. There is a severe shortage of trained mental health professionals for children and adolescents, with those living in low-and-middle income settings having the least support [3]. In spite of this burden, mitigation for mental illness, such as resources, programming, and system-level support, for children and adolescents remains a neglected priority.

This gap between the demand for mental health services and supply, which has existed long before the COVID-19 pandemic, poses a significant threat to the mental resiliency of our children and adolescents [4]. The COVID-19 pandemic has taken a dramatic toll across the globe and challenged families in unprecedented ways [5]. One study in the United States found that one in four parents reported that they were experiencing mental health deterioration and one in seven reported that their children’s behavioral health had worsened as a result of food insecurity in the home, loss of childcare services, and disruption in healthcare services [6]. Untreated mental health conditions existing prior to COVID-19 or those stemming from it can disrupt children and adolescents’ physical and mental functioning at school, at home, and in their communities [7]. Moreover, this distress can lead to extensive morbidity in the form of mental and substance-use disorders among children and adolescents [8]. Untreated diseases also increases the risk of self-harm, with suicide being the third leading cause of mortality among adolescents’ aged 15 to 19 years old [9]. It is evidenced that the rate of suicide among adults is high in low-and-middle income countries. Hence, there is a need to assess the risk of suicide among children and adolescents living in these countries to ensure that the burden of suicide is addressed with immediate action if needed [10].

Although significant evidence has been published regarding the physical impact of COVID-19 on the health of children and adolescents, there is a need for more extensive research on the present and future secondary health impacts as well as psychosocial implications. In terms of secondary effects, the World Vision’s COVID-19 Aftershocks Report estimated that 30 million children will be negatively affected by COVID-19 disruptions to the health care system, which will translate into increased rates of malnutrition, other preventable infectious diseases, and an inability to access essential vaccines [11]. The pandemic has also disrupted food systems and increased economic hardships on families, with many losing their jobs and unable to provide healthy meals for their children [12]. When the pandemic started, an analysis published in The Lancet estimated that an additional 6.7 million children under five years old could suffer from wasting due to food insecurity, with those children under the age of five in low-and middle-income countries being at highest risk for this suffering [13]. Inadequate nutrition can harm children’s emotional, cognitive performance, and personal development, all of which are related to mental health [14]. This risk disproportionately falls upon children in low-and-middle-income countries, who will only be further left behind if policies fail to prioritize them.

In terms of psychosocial impact, the challenge of social isolation on children and adolescents and their mental health must be addressed. An April 2020 UNICEF report found that nearly all children lived in a setting with some restriction on social mobility, 60% of children worldwide lived in countries under full or partial lockdowns, and an estimated 1.5 billion children had disrupted schooling [15]. With this restriction of movement and ability to engage as freely as before with support networks such as extended family or teachers, there was increased reporting of child abuse. Though services to address child abuse concerns were increasingly needed, 104 countries reported an interruption in services related to the protection of violence against children due to COVID-19 [16].

Factors such as social isolation, school closures and lockdowns, deaths of relatives, economic instability, and uncertainty about the future must be seriously considered as we assess the impact of COVID-19 on our children and adolescents [17]. Similar to the effects of war, violence, and poverty, COVID-19 has features ripe for generating transgenerational trauma that can lead to serious negative mental health consequences on children and adolescents given the traumatic toll that the pandemic has had on their parents. For example, studies conducted in Rwanda on the legacy of the 1994 Genocide against the Tutsi have shown that children and adolescents are affected by their parents’ post-traumatic disorder [18]. Some studies have shown that children and adolescents who were not even born at the time of these dramatic events were still affected [19].

At a systems-level, there was already a severe shortage of mental health care professionals worldwide prior to COVID-19 [20]. This shortage is worse when it comes to child and adolescent services where there is fewer than 0.1 child psychiatrists per 100,000 population in low-and-middle-income countries as compared to 1.19 per 100,000 population in high-income countries [21]. For those children with mental health conditions that predated the pandemic, their ability to obtain needed follow-up treatment such as medications or therapy was hindered due to disruptions in mental health services throughout the pandemic [22]. Without adequate health professional support, children and adolescents are forced to cope with conditions such as anxiety, loneliness, depression, and substance-use disorders – all of which were exacerbated by the pandemic and are associated with reduced quality of life during adulthood [23,24].

## Looking Ahead

Although far more research will be needed to capture the full extent of both near- and long-term psychological effects of COVID-19 on children and adolescents, we cannot wait to act. To effectively respond to the mental health needs of children and adolescents, we must hold health systems accountable to prioritize their mental health in both the ordinary as well as the extraordinary times. Governments and key community stakeholders must work together to implement sustainable solutions for children and adolescents’ mental crisis during this pandemic and beyond. Specific ideas include the provision of early diagnosis and treatment, educating caregivers on home-based interventions and community outreach services to protect and care for their children’s and adolescents’ mental health. A study conducted in Liberia to evaluate the implementation of community-based expressive arts programs for children and adolescents after the Ebola outbreak showed that focusing on expressive art activities such as play and yoga decreased mental stress symptoms among children and adolescents [25]. Moreover, other interventions such as the production of culturally adapted literary texts like *My hero is you: How Kids Can Fight COVID-19!* show the importance of storytelling and initiating family discussions to mitigate mental distress among children, help children understand the effects of the pandemic, and build their mental health literacy on how to deal with them [26]. Each of these efforts should build into the health and community systems so that they can be sustained as well as promote collaboration across sectors. Children and adolescents’ voices must be part of shaping these solutions.

To provide an example from Rwanda, under the patronage of the Ministry of Health in partnership with the Africa Centers for Disease and Control and the support of the World Health Organization, the University of Global Health Equity (UGHE) hosted the launch of the Children Depression Screening Tool (CDST) for early detection of depression for children living in stressful conditions and suffering from chronic diseases [27]. The CDST is a rapid, open-source, and easy-to-use screening tool created and validated in Rwanda to identify children ages seven to fourteen who are at risk of depression and refer them to mental health professionals for appropriate care [28]. The CDST was developed as an accessible tool that would ensure cultural adequacy in a mental health service provision. Although other tools exist, the CDST is age-specific, easily adaptable to other cultures by mental health practitioners and can be administered in five minutes by non-health professionals after a short training [27,28,29]. There is a plan to use the CDST in a school-based program in Rwanda, which is a useful example of a multisectoral home-grown solution that aims to improve mental health services for children and adolescents, not only in the COVID-19 era but beyond [27].

Too many children and adolescents have been suffering for far too long, and COVID-19 has only exacerbated and broadened this pain. In order to optimize their mental health, we must prioritize them now. We owe it to each one of them, wherever they live, to act intentionally, swiftly, and collectively as parents, community leaders, pediatricians, educators, mental health specialists, policymakers, investors, and all those who wish to improve their lives. Collectively – across all sectors of government (not only the health and education sectors), private institutions, and civil societies – we must invest and innovate sustainable solutions for the current and future mental health of our children and adolescents. This requires that we do not simply repair broken systems but reimagine a better way for delivering care to bolster the mental resiliency of our children and adolescents, now and for future generations.
